# Are some effector systems harder to switch to? In search of cost asymmetries when switching between manual, vocal, and oculomotor tasks

**DOI:** 10.3758/s13421-022-01287-1

**Published:** 2022-02-23

**Authors:** Mareike A. Hoffmann, Iring Koch, Lynn Huestegge

**Affiliations:** 1 Institute of Psychology, University of Würzburg, Röntgenring 11, 97070 Würzburg, Germany; 2 Institute of Psychology, RWTH Aachen University, Aachen, Germany

**Keywords:** Cognitive control, Task switching, Response modalities

## Abstract

In task-switching studies, performance is typically worse in task-switch trials than in task-repetition trials. These switch costs are often asymmetrical, a phenomenon that has been explained by referring to a dominance of one task over the other. Previous studies also indicated that response modalities associated with two tasks may be considered as integral components for defining a task set. However, a systematic assessment of the role of response modalities in task switching is still lacking: Are some response modalities harder to switch to than others? The present study systematically examined switch costs when combining tasks that differ only with respect to their associated effector systems. In Experiment 1, 16 participants switched (in unpredictable sequence) between oculomotor and vocal tasks. In Experiment 2, 72 participants switched (in pairwise combinations) between oculomotor, vocal, and manual tasks. We observed systematic performance costs when switching between response modalities under otherwise constant task features and could thereby replicate previous observations of response modality switch costs. However, we did not observe any substantial switch-cost asymmetries. As previous studies using temporally overlapping dual-task paradigms found substantial prioritization effects (in terms of asymmetric costs) especially for oculomotor tasks, the present results suggest different underlying processes in sequential task switching than in simultaneous multitasking. While more research is needed to further substantiate a lack of response modality switch-cost asymmetries in a broader range of task switching situations, we suggest that task-set representations related to specific response modalities may exhibit rapid decay.

## Introduction

Everyday life situations often confront us with successive demands, requiring us to switch between different tasks or actions. For example, navigating in traffic can involve switching from a situation in which we concentrate on lane keeping and driving with constant speed to depressing the brake pedal by foot and shifting gears in order to stop at a traffic light. Basic cognitive research has shown that such task switching comes at a cost, even when the only component of a task that changes is the response modality (e.g., switching from a manual to a pedal or vocal task). Under controlled conditions, that is, when successively executing the same task but with another (vs. the same) response modality, participants were shown to exhibit performance decrements (e.g., Philipp & Koch, 2011). The present study aims at studying such response modality switch costs systematically by focusing on oculomotor, manual, and vocal responses, and by looking for switch-cost asymmetries as potential markers for differences in task-set representation dynamics based on specific response modalities.

Traditionally, cognitive mechanisms of sequential task processing have been addressed using the task-switching paradigm (originally introduced by Jersild, 1927; see Kiesel et al., 2010; Koch et al., 2018; Monsell, 2003, for reviews), in which participants alternate between two (or more) tasks in short temporal succession. While Jersild (1927) originally simply compared overall performance in task blocks requiring task switches with that in task blocks involving the repeated execution of one and the same task, more recent studies utilize more sophisticated paradigms that allow for better conceptual specificity (e.g., by separately analyzing performance differences between single task blocks and task repetitions within mixing blocks, i.e., mixing costs, and performance differences between repetition and switch trials in mixing blocks, i.e., switch costs). These paradigms comprise the alternating-runs paradigm (involving predictable task switches, Rogers & Monsell, 1995), task cuing (involving unpredictable task switches indicated by cues, e.g., Fintor et al., 2019; Meiran, 1996; Sommer & Lukas, 2018; Sudevan & Taylor, 1987), or voluntary task switching (allowing participants to decide for themselves which task to execute, Arrington & Logan, 2004; recently used by, e.g., Fröber & Dreisbach, 2017; Jurczyk et al., 2018; Mittelstädt et al., 2018; see Arrington et al., 2014, for a review).

All these task-switching paradigms have in common that an alternation from one task to another is associated with performance decrements: Performance in task alternation trials is usually characterized by greater response times (RTs) and/or higher error rates than performance in task repetition trials. Thus, the difference in performance between RTs/error rates in switch trials and repetition trials represents switch costs. These switch costs are assumed to be based on the cognitive activation/inhibition of (and potential interference between competing) task sets in working memory (e.g., Koch et al., 2010). Such task sets are typically defined as the cognitive representations of task requirements including intentions, stimuli, potential responses and their modalities, as well as the mappings of stimuli to responses (e.g., Monsell, 1996, 2003; Rogers & Monsell, 1995; Vandierendonck et al., 2008). Crucially, it is assumed that during the execution of one task, some features of the other task are partially active, too, and can thereby affect performance. Thus, task-switching studies are particularly suited to address the dynamics and interactions of mental task representations.

More specifically, effects of persistent representations of the previous task set as well as processes of reconfiguration of the currently required task set are assumed to play a major role in task switching (see Kiesel et al., 2010) as potential origins of switch costs. Interestingly, these performance costs are often distributed *asymmetrically* among tasks, suggesting that the particular features of the task to be configured (or the task to be switched away from) matter for processing. Specifically, many studies reported that switching to a dominant (typically in the sense of better trained) task results in larger switch costs than switching to the less dominant task (e.g., Allport et al., 1994; Allport & Wylie, 1999; de Jong, 1995; Monsell et al., 2000; Yeung & Monsell, 2003). For example, Meuter and Allport (1999) observed asymmetric switch costs in the context of language switching: When bilingual participants switched between digit naming in their (better trained) first language versus their second language, they responded more slowly in their second (vs. first) language in repetition trials, but faster in their second (vs. first) language in switch trials (see, e.g., Declerck & Philipp, 2015, for a review; but see Gade et al., 2021, for limits regarding a generalization of these effects).

These findings provided important insights for the theoretical discussion of whether switch-cost asymmetries are mainly driven by inhibitory processes (differences in the amount of inhibition needed to suppress a currently irrelevant task set) or rather by differences in (re)configuration ease between two task sets (see Kiesel et al., 2010). However, in contrast to the assumption of a stronger inhibition of a better trained task set (e.g., Koch et al., 2010), the latter account would predict that switch costs should generally be lower when switching to well-learned (or dominant) tasks due to an easier (re)configuration (e.g., Meiran, 1996; Rogers & Monsell, 1995; Rubinstein et al., 2001). This is at odds with the observed switch-cost asymmetry effects reviewed above (e.g., Meuter & Allport, 1999). Thus, the observation that well-trained tasks are associated with particularly high switch costs rather supports the view that it is costly to reactivate a task set that had to be strongly inhibited previously to allow for an efficient execution of the less well-learned task in that previous trial (Allport et al., 1994; Koch et al., 2010; Meuter & Allport, 1999).

Crucially, Yeung and Monsell (2003) demonstrated that switch-cost asymmetries did not only rely on stronger or weaker task representations, but can also be affected by, for example, the assignment of response modalities to tasks (manual vs. vocal tasks). While this study did not specifically focus on differences between these response modalities but rather on the effect of the amount of response-set overlap, this observation represents one of the first hints that response modality-related task differences might also contribute to switch-cost asymmetries.

Even more relevant for the present study, Philipp and Koch (2010) demonstrated that merely switching among different response modalities (vocal and manual, vocal and foot, manual and foot) – while keeping all other task characteristics constant – already sufficed to yield switch costs. Such significant response modality switch costs were replicated in other studies for the case of switching between manual and foot responses (Hsieh et al., 2014; Philipp et al., 2013; see Table 1 for an overview of selected response modality switching studies). Philipp and Koch (2005) already speculated that the specific response modality required for executing a task could represent a relevant component of a task set, which explains why a change in response modality suffices to yield switch costs. Moreover, in one particular study (Philipp & Koch, 2011) first evidence for response modality-based switch-cost *asymmetries* was observed: When switching between vocal and manual responses in the context of an otherwise identical task, switching to the vocal response was associated with greater performance costs (using response modality repetitions as a baseline) than switching to the manual response. In contrast, no significant cost asymmetries in this study were reported for switching between vocal and foot responses, or for switching between manual and foot responses (see also Philipp et al., 2013). Finally, in a study by Lawo and Koch (2015) on auditory attention switching, no significant asymmetry of switch costs has emerged when comparing attention switching with vocal responses (in one block of trials) and with manual responses (in another block of trials). Note, however, that this latter study did not involve trial-by-trial switching of response modalities and should thus be interpreted with caution in the present context.

**Table 1:** Overview of selected studies involving trial-by-trial switches of response modality. Not included are studies in which response modality was manipulated block-wise (instead of trial-by-trial, e.g., see Lawo & Koch, 2015) or studies that only involved switches of responses within a single effector system (e.g., change of finger or hand for responding, see Hsieh et al., 2014, for example references)

Study	Task(s)	Response modality combination	CSI/RCI/RSI	Cue/stimulus modality	Significant modality switch costs	Modality switch-cost asymmetry?
Yeung & Monsell (2003, Exp. 3)	Digit naming/digit color judgment (predictable order without cues)	M-V (N = 8 in relevant “RCO” group)	RSI: 1000 ms	Stimulus: visual	75 ms (est.), but confounded with task switch cost	not analyzed
Philipp and Koch (2010)	Digit magnitude/digit parity judgment	M-V (N = 48/8/16 in Exp. 1/2/3) P-V (Exp. 2: N = 8) M-P (Exp. 2: N = 8)	CSI: 600 & RCI: 1000 ms (Exp. 1), CSI: 100/1000 ms (Exp. 2/3)	Cues: visual + auditory (only visual in Exp. 3) Stimulus: Visual	M-V: 336/288/400 (est.) ms in Exp. 1/2/3, P-V: 245 ms, M-P: 265 ms (averaged across CSI cond.)	not analyzed
Philipp & Koch (2011, Exp. 2)	Digit magnitude/digit parity judgment	M-V (N = 8) P-V (N = 8) M-P (N = 8)	CSI: 100 vs. 1000 ms RSI: 1600 ms	Cue: visual Stimulus: visual	M-V: 116 ms; P-V: 87 ms; M-P: 91 ms	sign. *only* in M-V group (vocal switch costs 8/70 ms larger than manual switch costs in CSI 100/1000 ms condition, resp.)
Hsieh et al. (2014)	Color/shape match-to-sample task (Exp. 1); color/shape judgment task (Exp. 2)	M-P (N = 24/16 in Exp. 1/2)	CSI: 2000 ms RCI: 1300 ms	Cue: visual Stimulus: visual	Exp. 1: 180 ms; Exp. 2: 191 ms	not analyzed
Philipp et al. (2013)	Color/shape judgment task	M-P (N = 23)	CSI: 200 ms RCI: 4500, 5000, 5500 ms	Cue: visual Stimulus: visual	88 ms	no significant asymmetry

*Note*. *CSI* cue-stimulus interval, *RCI* response-cue interval, *RSI* response-stimulus interval

Response modality combinations: *M-V* manual-vocal, *P-V* pedal-vocal, *M-P* manual-pedal

Mean modality switch costs in some studies had to be estimated (“est.”) based on information depicted in graphs

Taken together, we can conclude that although the noteworthy observation of a significant response modality cost asymmetry in Philipp and Koch (2011) was only a side observation made in a comparatively small sample of participants, this may represent another hint that particular task characteristics related to response modalities can potentially give rise to switch-cost asymmetries (e.g., Allport & Wylie, 1999; de Jong, 1995; Meuter & Allport, 1999; Yeung & Monsell, 2003). However, given the lack of a clear empirical consensus (partly also due to the fact that some relevant studies did not report switch costs separately for each modality, e.g., Hsieh et al., 2014; Philipp & Koch, 2010), the issue of response modality-based switch-cost asymmetries still appears to be unresolved and calls for further attention.

The idea of cost asymmetries based on different effector systems is strengthened by cumulative evidence from recent years suggesting a consistent prioritization pattern across tasks only differing in their involved response modalities (i.e. effector systems) in *temporally overlapping* dual-task situations. Specifically, in dual-task studies, Huestegge and Koch (2013) and Hoffmann and colleagues (Hoffmann et al., 2019) demonstrated that dual-task costs (calculated by subtracting mean RTs in single-task blocks from corresponding mean RTs in dual-task blocks) due to multiple action demands follow an asymmetrical pattern: Oculomotor responses are associated with the smallest dual-task costs followed by pedal responses, vocal responses, and finally manual responses (see also Hoffmann, Westermann, et al., 2020; Pieczykolan & Huestegge, 2014). These findings have been interpreted as evidence for an influence on central capacity allocation schemes based on task prioritization rooted in the particular response modalities associated with a task. While the functional significance of this effector prioritization pattern has remained elusive, some explanatory accounts have previously been discussed. For example, one might speculate that inherent features of specific effector systems (such as the ballistic nature of saccades, which cannot be corrected after a certain “point of no return”) could explain a general prioritization of such actions in order to maximize overall task performance (e.g., Pieczykolan & Huestegge, 2014). Another possible explanation is that the prioritization of specific response modalities could have evolved based on hereditary evolutionary advantages, for example, by prioritizing the gathering of visual information (by moving one’s eyes) or running away over responding vocally or manually to potentially life-threatening situations. However, previous data and explanations on effector prioritization focused on simultaneous action processing only, while in task switching such a systematic examination of a potential effect of this type of response modality-based costs asymmetries is not available. Yet, such a study would be highly informative regarding the underlying dynamics of modality-specific task-set representations in task switching.

In the present study, we compared switch costs (as well as mixing costs) between oculomotor and vocal (Experiment 1) and oculomotor, vocal, and manual responses (Experiment 2) in a cued task-switching paradigm. Our selection of particular tasks was guided by prior work regarding response modality-based task prioritization effects in temporally overlapping dual-task paradigms (e.g., Hoffmann et al., 2019; Huestegge & Koch, 2013), as these tasks produced very robust dual-task cost asymmetries in simultaneous dual-task situations. Otherwise identical tasks involving oculomotor responses showed smaller dual-task costs than those involving vocal responses, and tasks involving vocal responses showed smaller costs than those involving manual responses. If we interpret smaller dual-task costs in terms of task dominance (similar to task dominance based on training as, e.g., observed in language switching studies) and transfer these ideas to a typical task switching setting (see Hirsch et al., 2018, for evidence of shared processes underlying dual-task costs and mixing/switch costs), this should translate to greater switch costs when switching towards a task involving a prioritized response modality (i.e., oculomotor > vocal > manual). This would thus correspond to higher switch costs towards oculomotor responses than towards vocal (in both experiments) or manual tasks (in Experiment 2), and higher vocal than manual task switch costs (in Experiment 2, similar to the preliminary observations of Philipp & Koch, 2011). If, however, previously observed switch-cost asymmetries when switching between vocal and manual responses (Philipp & Koch, 2011) can simply be explained by differences in response latency speed (as vocal responses were associated with higher switch costs but also with significantly slower overall response speed), we should observe smallest switch costs in oculomotor responses that are typically executed much faster than responses in any other modality.

Finally, it is also possible that different dynamics of task-set representation characteristics contribute to a more nuanced pattern of results: For example, it is possible that specific effector system characteristics of a task dissipate quicker than, for example, language-related features, so that only general response modality-based switch costs may be observed, but no substantial switch-cost asymmetry.

## Experiment 1: Switching between oculomotor and vocal tasks

### Method

#### Participants

A power analysis (using the smallest partial eta-square = .30 regarding the effector-based dual-action cost asymmetry between oculomotor and vocal responses reported in Huestegge and Koch (2013), with an alpha of 5% and a power of 95%) revealed an optimal sample size of ten participants. As effects might be a bit smaller in the present task switching context, we decided to test 16 participants. All were naïve regarding the purpose of the study and gave informed consent. All participants were recruited from the local university’s student panel and received monetary reward or course credit. The threshold of chance performance level was equivalent to 41.0% errors regarding single task blocks, and equivalent to 44.8% errors regarding mixing blocks (calculations based on a binomial test specified by a chance level of 50%, alpha = 5% and usable number of trials amounting to 78 or 174, respectively). Based on these criteria we excluded and recollected data of two participants (to ensure full counterbalancing). The final sample consisted of four males and twelve females (15 right-handed) with a mean age of 27.1 years (*SD* = 6.3). All had normal or corrected-to-normal hearing and vision.

#### Apparatus and stimuli

Participants were seated approximately 67 cm in front of a 21-in. cathode ray tube screen. Spatial resolution was 1,024 × 768 pixels and temporal resolution amounted to 100 Hz. An eye-tracker sampling eye movements at 1,000 Hz (Eyelink 1000, SR Research Mississauga, Ontario, Canada) was utilized to register saccade latencies and amplitudes of the right eye to register oculomotor responses. Head movements were minimized by means of a chinrest. Vocal RTs were registered and logged by using the integrated voice key function of the programming software Experiment Builder (version 2.1.140, SR Research) via a microphone (Sennheiser e 835-S) in front of the participants. Experiment Builder was also used to run the experiment.

As imperative stimuli, we used 1,000-Hz sinusoidal tones presented either to the right or to the left ear via supra-aural headphones (Sennheiser, PMX 95). As visual task cues indicating the required response modality, we used small schematic pictures of an eye (height 0.86°, width 1.45° visual angle, indicating oculomotor response) or a mouth (height 0.68°, width 1.97° visual angle, indicating vocal response) presented at the location of the fixation cross.

Throughout each block, a white fixation cross (size = 0.43° of visual angle) at the center of a black background and two white rectangular squares at an eccentricity of 8.5° of visual angle (size = 0.43° each) to the left and right of the central fixation cross remained present on the screen. These white rectangular squares served as spatial targets for oculomotor responses: For instance, when a right oculomotor response was required participants were instructed to look at the right target square (and to redirect their gaze to the central fixation cross afterwards). The vocal task was to utter the words “links” or “rechts” (German for “left”/“right”) in a spatially congruent manner with respect to the presentation side of the stimuli.

#### Procedure

At the beginning of the experiment (as well as at the beginning of each block) participants received instructions verbally from a research assistant and in written format via an instruction screen. The experiment consisted of ten blocks, always starting with two single task blocks consisting of 40 trials each, in which participants should either execute the vocal task or the oculomotor task throughout the block. Which response modality was required in the first (vs. the second) block was counterbalanced across participants. Afterwards, all participants underwent one training block of the mixing condition (consisting of 20 trials) followed by five response-modality mixing blocks (60 trials each). In the end, the two single task blocks were repeated in the same order as at the beginning of the experiment.

Irrespective of block type, each trial began with the presentation of a visual cue. In single task blocks, the respective visual cue was also presented. Thus, in single-vocal blocks there were only mouth cues, and in single-oculomotor blocks there were only eye cues, while the cue type switched randomly in training blocks as well as response modality mixing blocks. After a cue-stimulus interval (CSI) of 200 ms, the imperative auditory stimulus was presented for 80 ms. 1,100 ms after the registration of the response the next trial started with the presentation of a cue (response-cue interval, RCI). Whenever no response was registered within 4 seconds after stimulus presentation, the procedure automatically proceeded with the next trial. Visual cues remained on screen until response execution (or until the beginning of the next trial after 4 s). Saccades were counted as responses when their amplitude was greater than 3° visual angle (corresponding to half of the distance between central fixation cross and peripheral target stimuli).

#### Design

The independent variables were response modality (oculomotor vs. vocal) and response modality transition (single task vs. repetition vs. switch). The dependent variables were RTs (in ms) and error rates (percentages). Data analyses were separated into two (non-orthogonal) contrast analyses. In the switch-cost contrast analysis, we included switch and repetition trials in the mixing block, whereas in the mixing-cost contrast analysis, we included repetition trials from the mixing block and the single task trials (which represent repetition trials by definition).

### Results and discussion

Data from training blocks were not considered in the analyses. Also, the first trial of single-modality blocks and the first two trials of modality mixing blocks were discarded. Furthermore, all trials in which either no response, an oculomotor response with a latency below 50 ms, or a vocal response with a latency below 200 ms was registered were defined as invalid (2.4%) and excluded from all further analyses to ensure that, for example, voice key artifacts (that do not represent intended responses) do not distort the data. Moreover, all trials in which the first registered response was executed in the wrong modality (e.g., a saccade although a vocal response was required) were excluded (7.7%). We decided to treat those trials as invalid because thresholds and definitions for counting as a saccade response or vocal response cannot be perfectly comparable between effector systems, rendering a clear interpretation difficult. All trials following trials in which not the required response was executed (because no response at all was registered or due to an invalid response in the wrong modality) had to be excluded because they cannot be unequivocally interpreted as switch or repetition trials. Directional errors (5.2% of valid trials, e.g., looking right instead of left) were not included in RT analyses.

Means and SDs of RTs and error rates (ERs) are summarized in Table 2. A 2 × 3 ANOVA regarding RTs revealed a significant main effect of response modality: Oculomotor responses (248 ms) were executed faster than vocal responses (664 ms), *F*(1, 15) = 526.95, *p* < .001, η^2^_p_ = .97. There was also as a significant main effect for response modality transition *F*(1, 15) = 39.68, *p* < .001, η^2^_p_ = .73. Pairwise planned contrast analyses revealed significant overall mixing costs of 43 ms (comparison of single task trials and repetitions within mixing blocks), *F*(1, 15), 14.31, *p* = .002, η^2^_p_ = .49, and significant overall switch costs of 56 ms (comparison of repetition trials vs. switch trials within mixing blocks), *F*(1, 15) = 62.93, *p* < .001, η^2^_p_ = .81. However, there was no interaction of response modality and response modality transition in RTs, *F*(1, 15) = 0.10, *p* = .901, η^2^_p_ = .01. Thus, there were no significant differences in switch costs or mixing costs between the two tasks involving different response modalities (Table 3).

**Table 2:** Mean response times (RTs) and error rates (+SDs) for oculomotor vs. vocal responses in single task blocks as well as for repetition and switch trials in mixing blocks

	RTs (in ms)		ERs (in %)	
Condition	*M*	*SD*	*M*	*SD*
Oculomotor				
Single	203	42	3.4	3.1
Repetition (mixing blocks)	245	46	6.7	5.9
Switch (mixing blocks)	297	66	9.6	8.1
Vocal				
Single	615	90	0.5	1.1
Repetition (mixing blocks)	659	88	2.2	3.3
Switch (mixing blocks)	720	94	4.4	4.6

**Table 3:** Mean (+SDs) oculomotor vs. vocal mixing costs and switch costs in response times (RTs) and error rates (ERs) plus statistical test results of corresponding t-test comparisons

	Mixing costs				
	Oculomotor	Vocal			
	*M (SD)*	*M (SD)*	*t*(15)	*p*	*d*
RTs (ms)	42 (50)	44 (68)	0.13	.902	0.04
ERs (%)	3.3 (5.3)	1.7 (3.7)	1.13	.277	0.36
	Switch costs				
	Oculomotor	Vocal			
	*M (SD)*	*M (SD)*	*t*(15)	*p*	*d*
RTs (ms)	53 (41)	61 (72)	0.31	.764	0.14
ERs (%)	2.9 (7.7)	2.3 (5.1)	0.41	.681	0.09

The same 2 × 3 ANOVA on ERs revealed a significant main effect of response modality, showing more oculomotor (6.5%) than vocal errors (2.2%), *F*(1, 15) *=* 15.14, *p* = .001, η^2^_p_ = .50. There was also a significant main effect of response modality transition, *F*(1, 15) *=* 9.00, *p* = .001, η^2^_p_ = .36. Planned comparison analyses of single-task trials with repetitions in mixing blocks and of repetitions and switches within mixing blocks revealed significant overall mixing costs of 2.2% in ERs, *F*(1, 15) 8.03, *p* = .013, η^2^_p_ = .35. There was no significant difference between repetition (4.4%) versus switch trials (7.0%) within mixing blocks (but showing the same trend towards switch costs as in RT), *F*(1, 15) = 3.21, *p* = .094, η^2^_p_ = .18, and again no interaction of response modality and response modality transition in ERs, *F*(1, 15) = 1.23, *p* = .307, η^2^_p_ = .08, which shows that also regarding ERs neither mixing costs nor switch costs differed between the two tasks involving different response modalities (Table 3).

Experiment 1 revealed significant response modality mixing costs as well as response modality switch costs when switching between an oculomotor task and a vocal task. This observation supports our hypothesis derived from previous studies (Hsieh et al., 2014; Philipp et al., 2013; Philipp & Koch, 2010, 2011) that response modalities are an integral component of a task set, because an alternation of the response modality (while keeping all other task components constant) yielded typical effects associated with task alternations, namely mixing costs and switch costs. Note that unlike these previous studies we combined a vocal task and an oculomotor task, and used auditory (instead of visual) stimuli (which was only done in Lawo & Koch, 2015, but without implementing response modality switches within blocks). Therefore, the present findings extend these previous results, and we can also exclude that the emergence of response modality mixing costs and response modality switch costs is restricted to visual stimulation conditions.

Interestingly, however, we did not observe any significant modulation of mixing costs or switch costs as a function of response modality. There was neither any performance advantage nor disadvantage for either task set (in terms of larger/smaller oculomotor vs. vocal switch costs), and therefore no indication of task dominance (as defined in terms of switch-cost asymmetries). Thus, our results support neither the assumption of oculomotor tasks being dominant (as was discussed in the context of switch-cost asymmetries related to language switching) and therefore associated with substantially greater switch costs due to stronger inhibition, nor any theoretically meaningful switch-cost asymmetries simply due to different RT baselines.

Of course, we replicated well-known general effects such as faster and more error-prone responses in the oculomotor versus vocal domain, which highlight the typical differences between these particular response systems (e.g., Hoffmann et al., 2019). However, the dual-task cost asymmetries observed in simultaneous multitasking paradigms (Hoffmann et al., 2019) – probably indicative of effector-system based task prioritization – clearly did not show up in a comparable way in the present task-switching paradigm.

Taken together, these results indicate that response modality switching can indeed be seen as a form of task switching, but they also show that response modality-based cost asymmetry effects observed in simultaneous multitasking paradigms (e.g., Hoffmann et al., 2019; Huestegge & Koch, 2013) do not readily translate to corresponding substantial effects in task switching. However, these observations from Experiment 1 are still restricted to a narrow range of response modalities (combination of vocal and oculomotor tasks). Therefore, and prior to speculating further about the reasons for the lack of a significant cost asymmetry effect in mixing costs and switch costs, we conducted Experiment 2, in which we extended our setting to a third response modality. Specifically, we compared switch costs and mixing costs between oculomotor, vocal, and manual responses because manual RTs are typically located between the RT levels of the other two modalities, while at the same time representing the response modality at the extreme (low) end of the prioritization order proposed by Hoffmann et al. (2019). Moreover, given the null effect for the interactions in Experiment 1, we increased the sample size in Experiment 2 very substantially to increase statistical power for detecting any potentially meaningful dominance effect (if at all present) on mixing costs and switch costs. Based on these two new design features, Experiment 2 should be suited to more coherently answer the question of whether previously observed switch-cost asymmetries based on response modality switches in simultaneous dual tasks can or cannot be observed also in sequential task switching.

## Experiment 2: Switching among oculomotor, vocal, and manual responses

As the findings of Experiment 1 indicated that there were no indications in performance measures for any switch-cost asymmetry between oculomotor and vocal tasks, we were interested to take a closer look at potential effects when combining other response modalities across tasks. Thus, in Experiment 2 we systematically compared mixing costs and switch costs among pairwise combinations of tasks involving vocal, oculomotor, and manual responses in one integrative within-subject design. With this approach we should be able to ultimately answer the question of whether previously reported greater switch costs for vocal than for manual responses are driven by differences in response latencies between tasks, by a stronger inhibition associated with the vocal tasks, or whether they represent a special case that only holds for this specific response modality combination. We again built on the tasks that were already shown to be suited to generate substantial dual-task cost asymmetries in simultaneous dual tasking to maximize the potential for observing similar effects in task switching.

Note that results from Experiment 1 already speak against a simple *explanation in terms of response latencies*, as the difference between vocal and oculomotor response latencies is even greater than that between vocal and manual responses (e.g., see Hoffmann et al., 2019). The *response modality-based task prioritization explanation*, in contrast, would predict greater oculomotor switch costs compared to manual switch costs and greater vocal switch costs compared to manual switch costs (plus potentially greater oculomotor switch costs compared to vocal switch costs, even though a corresponding effect did not show up in Experiment 1), resulting from a stronger persistent inhibition for tasks that are located at a “higher” position within the ordinal prioritization pattern oculomotor > vocal > manual known from dual-task studies (see Hoffmann et al., 2019). Although such observations could not ultimately answer why we did not observe greater oculomotor switch costs than vocal switch costs in Experiment 1, it would still strengthen an overall explanation similar to that in the case of language switching (referring to some kind of task dominance). If, however, we do not observe any clear differences in switch costs among these tasks with different response modalities, this would speak against both accounts. In particular, such a lack of switch-cost asymmetries would rather indicate a quick decay of effector-related prioritization representations, so that this particular type of effector-based dominance mainly comes into play when capacity needs to be scheduled among *concurrent* tasks, but not in the same way in situations with sequential, *alternating* tasks.

### Method

#### Participants

Considering the results of Experiment 1, we decided to collect data of a relatively large sample to minimize the risk of finding null effects due to low statistical power (see power analysis in Experiment 1 for details). Therefore, and due to counterbalancing constraints, we decided to collect the data of 72 new participants. Following the same rationale as in Experiment 1, we excluded participants who did not perform better than chance level (> 41.0% errors in single blocks, > 43.7% errors in mixing blocks) to ensure that all participants followed task instructions. Based on this criterion, eight participants had to be excluded. One further participant aborted the experiment. We replaced these data with that of nine new participants to ensure full counterbalancing of our design (see below). The final sample consisted of 52 females and 20 males, with a mean age of 26.2 years (*SD* = 9.1). All participants were right-handed. Again, all gave informed consent, had normal or corrected-to-normal hearing and vision, and were rewarded monetarily or by course credit.

#### Apparatus, stimuli, and procedure

Apparatus and stimuli were the same as in Experiment 1. However, since here manual responses were also required, a visual cue indicating manual key-press responses was additionally included (a small hand, height 1.54°, width 1.71° visual angle). Key-presses were registered using a standard (German) QWERTZ keyboard on which the relevant keys (arrow left for left responses, arrow right for right responses, operated by the right index finger) were marked by two green stickers. Participants were instructed to leave their right index finger loosely on the arrow down key as a “home key position” when no manual response was required. Key-presses and manual response latencies were registered by the Experiment Builder software.

Similar to Experiment 1, Experiment 2 always started and ended with three single-task blocks (40 trials) for all (here: three) relevant response modalities. Again, the sequence of these response modalities was counterbalanced across participants but remained constant (i.e., was repeated in the same order at the end of the experiment) within individuals. The middle part of the experiment consisted of 12 blocks, one training block (20 trials) and three mixing blocks (60 trials) for each pairwise combination of response modalities (oculomotor-vocal, oculomotor-manual, vocal-manual). The order of pairwise combinations was counterbalanced across participants. Visual cues indicating which response modality was required were again randomized in training and mixing blocks. All other details were the same as in Experiment 1.

#### Design

Independent variables were response modality (oculomotor vs. vocal vs. manual) and response modality transition (single task vs. repetition vs switch). The dependent variables were RTs (in ms) and error rates (percentages). Similar to Experiment 1, our main research question is reflected in the two non-orthogonal contrast analyses of switch costs (switch trials vs. repetition trials in the mixing block) and of mixing costs (repetition trials from the mixing blocks vs. single task trials).

### Results and discussion

We used the same rationale to define invalid or erroneous trials as in Experiment 1. Again, the first trial in single task blocks and the first two trials in mixing blocks were not included in the analyses. Trials in which no response was registered (1.6%) and trials in which the voice-key trigger registered a sound but no word was uttered or in which another key (other than the left or right arrow key) was registered as a response (0.3%) were defined as invalid and discarded. Responses within 50 ms (regarding oculomotor responses) or within 200 ms (regarding vocal or manual responses) were discarded, too, to exclude measurement artifacts (1.0%). Again, all trials in which the (first) registered response was executed in the wrong response modality were excluded (further 6.4%). Lastly, all trials following trials in which no response in the required modality was executed (i.e., those that cannot be interpreted as either switch or repetition trials) were excluded (resulting in 84% valid trials altogether). Directional errors (4.4% of valid trials) were not included in RT analyses. Means and SDs of RTs and ERs are summarized in Table 4.

**Table 4:** Mean (+SDs) response times (RTs) and error rates (ERs) of oculomotor, vocal, and manual tasks in single modality blocks as well as in repetition and in switch trials in modality mixing blocks

	RTs in ms		ERs in %	
Condition	*M*	*SD*	*M*	*SD*
Oculomotor				
Single	231	48	7.6	7.3
Repetition (mixing blocks)	283	66	7.6	9.3
Switch (mixing blocks)	342	79	11.1	11.2
Vocal				
Single	641	99	1.2	3.5
Repetition (mixing blocks)	705	92	1.7	2.0
Switch (mixing blocks)	779	135	3.5	4.0
Manual				
Single	428	93	1.4	1.8
Repetition (mixing blocks)	502	108	3.3	4.3
Switch (mixing blocks)	557	144	4.4	6.6

Conceptually similar to Experiment 1, we (now) conducted a 3 × 3 ANOVA and two planned contrast analyses to examine mixing costs and switch costs regarding RTs and ERs. In case of sphericity violations, Greenhouse-Geisser corrections were used and uncorrected degrees of freedom and respective ɛ estimates are reported.

**Fig. 1:**
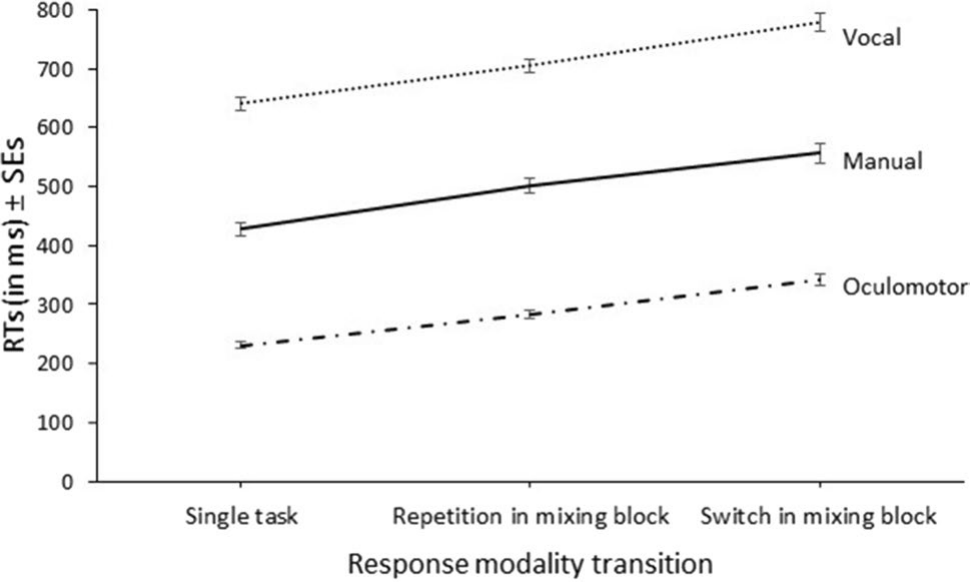
Response times (RTs) (± standard errors of the mean) for vocal, manual, and oculomotor responses in single-task trials as well as in repetition and switch trials in mixing blocks

RTs among the different response modalities and response modality transition conditions are illustrated in Fig. 1. The corresponding 3 × 3 ANOVA revealed a significant main effect of response modality, *F*(2, 142) = 1084.49, *p* < .001, η^2^_p_ = .94. Post hoc contrasts revealed that oculomotor responses (285 ms) were executed overall faster than vocal responses (708 ms), *F*(1, 71) = 1938.57, *p* < .001, η^2^_p_ = .97, and manual responses (496 ms), *F*(1, 71) = 565.85, *p* < .001, η^2^_p_ = .89, while manual responses were executed faster than vocal responses, *F*(1, 71) = 587.33, *p* < .001, η^2^_p_ = .89. Also, the main effect of response modality transition, comparing single-task trials (433 ms), repetition trials in mixing blocks (497 ms), and switch trials in mixing blocks (559 ms), was significant, *F*(2, 142) = 227.38, *p* < .001, η^2^_p_ = .76, ɛ = .69. The interaction of response modality and response modality transition was significant, too, *F*(4, 284) = 2.90, *p* = .035, η^2^_p_ = .04, ɛ = .76.

The contrast analyses revealed significant overall mixing costs of 63 ms, *F*(2, 142) = 187.01, *p* < .001, η^2^_p_ = .73, as well as significant overall switch costs of 63 ms, *F*(2, 142) = 157.74, *p* < .001, η^2^_p_ = .69.

Mixing costs differed among response modalities, *F*(2, 142) = 3.71, *p* = .027, η^2^_p_ = .05. Paired *t*-test comparisons showed that oculomotor mixing costs (52 ms) were significantly smaller than manual mixing costs (74 ms), *t*(71) = 2.91, *p* = .005, *d* = 0.41, but neither of them differed significantly from vocal mixing costs (64 ms), *p*s > .12. In contrast, switch costs did not differ significantly between the tasks with different response modalities, as indicated by a non-significant interaction of response modality and response modality transition, *F*(2, 142) = 2.58, *p* = .079, η^2^_p_ = .04. Numerically, there was a small trend towards greater vocal switch costs (75 ms) than manual switch costs (55 ms), while oculomotor switch costs (59 ms) were at an intermediate level^1^.

Errors occurred relatively rarely (4.4%). Nevertheless, the same 3 × 3 ANOVA regarding ERs revealed significant main effects of response modality, *F*(2, 142) = 54.87, *p* < .001, η^2^_p_ = .44, ɛ = .68, and of response modality transition, *F*(2, 142) = 15.95, *p* < .001, η^2^_p_ = .18, ɛ = .74, showing overall differences between oculomotor (8.7%), vocal (2.1%), and manual (3.1%) errors as well as between single-task trials (3.4%), repetition trials in mixing blocks (4.2%), and switch trials in mixing blocks (6.3%). Post hoc contrasts revealed significant differences among the response modalities in all pairwise comparisons (oculomotor vs. vocal: *F*(1, 71) = 63.84, *p* < .001, η^2^_p_ = .47, oculomotor vs. manual: *F*(1, 71) = 57.01, *p* < .001, η^2^_p_ = .46, vocal vs. manual: *F*(1, 71) = 5.77, *p* = .019, η^2^_p_ = .08). Regarding response modality transition, post-hoc pairwise comparisons revealed differences between single task trials and switch trials in mixing blocks, *F*(1, 71) = 18.58, *p* < .001, η^2^_p_ = .21, and between repetition trials in mixing blocks and switch trials in mixing blocks (i.e., switch costs), *F*(1, 71) = 22.48, *p* < .001, η^2^_p_ = .24, but not between single task trials and repetition trials in mixing blocks (i.e., mixing costs), *F*(1, 71) = 3.07, *p* = .084, η^2^_p_ = .04. However, there was no interaction of response modality and response modality transition, *F*(4, 284) = 2.32, *p* = .080, η^2^_p_ = .03, ɛ = .71 in ERs.

In sum, the results of Experiment 2 replicated the findings of Experiment 1 as well as a central result reported in Philipp and Koch (2011): An alternation of the response modality (while keeping other task features constant) yielded effects similar to those observed in more typical task switching settings (e.g., those in which task switches were defined in terms of a switch in S-R mapping rules), namely significant mixing costs and switch costs. However, while on a descriptive level vocal switch costs were a bit larger than manual switch costs in RTs (in line with previous observations of Philipp & Koch, 2011), this trend failed to reach statistical significance. Unlike the data set in Philipp and Koch (2011), which involved only a small sample, the present experiment is characterized by quite substantial statistical power. Taken together, all currently available evidence therefore suggests that if an effector-based switch-cost asymmetry effect between manual and vocal tasks is real, it is likely of very small size.

This view is further corroborated by the fact that regarding oculomotor responses we again observed no systematic differences in switch costs when compared to the tasks involving the other two response modalities included in this experiment. Numerically, the difference between oculomotor and vocal switch costs even pointed in the direction of *smaller* switch costs for the oculomotor system. Thus, tasks involving oculomotor responses, which were frequently shown to be associated with smaller dual-task costs in previous studies involving *simultaneous* multiple action control (e.g., Hoffmann et al., 2019; Huestegge & Koch, 2013; Pieczykolan & Huestegge, 2014) were clearly not associated with smaller switch costs in a task switching setting. At first sight, this appears to speak against the general idea that task sets involving oculomotor responses are in some way prioritized over those involving other response modalities. However, an alternative explanation related to the representational dynamics of specific task set characteristics is also possible and will be further elaborated in the *General discussion*.

## General discussion

The aim of the present study was to investigate response modality switching and potential effects of response modality-based task dominance on performance in task switching. By having participants switch between tasks that only differ in their associated effector system (oculomotor, vocal, manual), we tested whether effects typical for task switching occur (in particular, switch costs and mixing costs), and whether switch-cost asymmetries can be observed. In Experiment 1, participants switched between oculomotor and vocal tasks that were otherwise comparable (i.e., they involved the same basic left/right spatial response task). In Experiment 2, participants switched between oculomotor and vocal, oculomotor and manual, and vocal and manual tasks (all pairwise combinations were implemented within participants).

In both experiments, we observed reliable mixing costs and switch costs, suggesting that the alternation of the response modality (under otherwise constant task requirements) sufficed to negatively affect task performance. Thereby, we were able to replicate and extend previous findings reported by several studies (e.g., Hsieh et al., 2014; Philipp et al., 2013; Philipp & Koch, 2010; Philipp & Koch, 2011) suggesting that typical task-switching effects can also be observed by merely changing response modalities associated with the task. Note that these previous studies involved different task-switching setups, specific task requirements, and response modality combinations to demonstrate these effects of response modality switching. For example, Philipp and Koch (2011) used a magnitude or parity numerical judgment task instead of a spatial left/right task, and visual instead of auditory stimuli. Thus, the present results demonstrate that corresponding effects are reliable and generalize to a variety of tasks and stimulus conditions. In sum, these observations suggest that response modalities are indeed an integral component of a task set, irrespective of the particular type of task or task-switching setup.

Interestingly, none of the present experiments revealed any significant switch-cost asymmetries when switching between tasks involving different effector systems, neither in RTs nor in ERs. This pattern of results does therefore not readily corroborate a previous observation in Philipp and Koch (2011), who reported evidence for greater vocal than manual switch costs. Nevertheless, Experiment 2 revealed a numerical trend towards such an effect, thereby warranting a more elaborate discussion. It is important to note that Philipp and Koch (2011) used visual instead of auditory imperative stimuli. At first sight, one might therefore speculate that this difference might probably explain the lack of effects in the present study, especially as previous research has indicated that the particular pairings of input and output modalities in the two tasks can substantially affect switch cost patterns (e.g., Fintor et al., 2019; Fintor et al., 2020; Fintor et al., 2018; Hazeltine et al., 2006; Stephan & Koch, 2011, 2015). However, a closer look at this literature reveals that it is especially advantageous for vocal output when it is triggered by auditory input. As a consequence, our present research design – by implementing auditory (instead of visual) input – should, if anything, have made the vocal task even more dominant. It thus appears unlikely that the differences in input modalities between studies could explain the differences in observed effects. Moreover, as we observed robust response modality switch costs in the first place (see above), an important precondition for observing potential asymmetries in these costs was clearly established. Thus, the lack of response modality switch-cost asymmetries cannot simply be explained by a lack of meaningful costs to begin with.

Instead, it appears more likely that if an effector-based switch-cost asymmetry effect between manual and vocal tasks is real, it is likely of very small size (especially in the light of the fact that Experiment 2 was highly powered) and dependent on minute details of particular task characteristics. This view is further strengthened by the fact that we did not find any evidence for oculomotor dominance (in terms of corresponding asymmetric switch costs) in any of the two experiments, despite the fact that oculomotor tasks have been repeatedly shown to exhibit strong dual-task cost asymmetries (as reflected in large effect sizes) when combined with similar tasks involving other effector systems in simultaneous dual tasking (e.g., Hoffmann et al., 2019; Huestegge & Koch, 2013).

Even though at least Experiment 2 was designed to be highly powered, the absence of any clear switch-cost asymmetries (either similar to switch-cost asymmetries observed in language switching studies, Meuter and Allport (1999), or similar to effector-based dual-task cost asymmetries, Hoffmann et al. (2019)) essentially represents a null effect. Principally, it is of course possible that still more power is needed to observe corresponding switch-cost asymmetries. To assess this possibility more directly, we analyzed the 95% CI of the switch-cost asymmetries observed in our present study and compared the results with the effects reported in Meuter and Allport (1999), Hoffmann et al. (2019), and in Philipp and Koch (2011). In Experiment 1, we expected to observe greater oculomotor than vocal switch costs. However, the data revealed a reversed pattern, and the 95% confidence interval (CI) [-62; 47] neither contained the size of the switch-cost asymmetry reported in Meuter and Allport (1999), which amounted to 58 ms, nor the size of the corresponding effector-based dual-task cost asymmetry reported in Hoffmann et al. (2019), which amounted to 220 ms. The same holds for the 95% CI of the vocal-oculomotor switch-cost asymmetry in Experiment 2 (95% CI [-35; 4]). The manual-oculomotor switch-cost asymmetry (95% CI [-15; 22]) and the manual-vocal switch-cost asymmetry (95% CI [-3; 35]) were at least pointing in the expected direction, but the 95% CI never included the effect size for the corresponding dual-task cost asymmetries observed in Hoffmann et al. (2019), which amounted to 245 ms and 41 ms, respectively, or in Philipp and Koch (2011), where the corresponding significant cost asymmetry in the most comparable “long CSI” condition amounted to 70 ms. Taken together, this analysis of CIs unequivocally shows that the failure to observe a switch-cost asymmetry (comparable in size to either classic switch-cost asymmetries or effector-based dual-task cost asymmetries) cannot simply be ascribed to insufficient statistical power. Finally, it should also be kept in mind that a lack of response modality-based switch-cost asymmetries was repeatedly reported by a number of studies (Philipp et al., 2013; Lawo & Koch, 2015; two response modality combinations in Philipp & Koch, 2011), while reports of significant cost asymmetries are restricted to one small-sample observation only (one out of three response modality combinations in Philipp & Koch, 2011).

Thus, response modality-based differences in task sets do not exert a similar effect on sequential task switching as other types of task differences, such as those shown in the context of language switching. Those studies supported the interpretation that a stronger persistent inhibition of a dominant (more well-trained) task when it is currently not relevant causes higher performance costs when participants switch back to this dominant task (Allport et al., 1994; Allport & Wylie, 1999; de Jong, 1995; Meuter & Allport, 1999; Monsell et al., 2000; Yeung & Monsell, 2003). A corresponding rationale for response modality-based task processing differences (based on previously observed patterns in simultaneous dual tasking) would have predicted that switch costs for the oculomotor task should be particularly high, a finding that was clearly not present in our data. One possible explanation of this discrepancy is to assume that the mechanisms underlying response modality-based task prioritization in simultaneous dual tasking (Hoffmann et al., 2019) cannot be transferred to task switching (e.g., Allport & Wylie, 1999; Meuter & Allport, 1999; Monsell et al., 2000; Yeung & Monsell, 2003). Instead, the results might point towards different underlying prioritization mechanisms for multitasking situations with and without temporal task overlap.

### Why no response modality-based switch-cost asymmetry?

In our view, the most probable explanation for the lack of asymmetrical switch costs in the present study (as opposed to, e.g., language switching studies) is the assumption of different representational dynamics of certain task set features. Specifically, it is possible that effector system representations in task sets dissipate quicker than, for example, language-related features, so that only general response modality-based switch costs can be observed, but no (strong) switch-cost asymmetry. In contrast, a longer lasting activation of language-related task features could explain why asymmetrical task switch costs are typically found in studies involving language switching (e.g., Meuter & Allport, 1999). Nevertheless, response modality-based task prioritization processes may still come into play when such differences in representational dynamics do not matter, in particular in situations when both tasks need to be coordinated simultaneously. This could then explain why strong response modality-based cost asymmetries can be observed in simultaneous dual tasking (as in Hoffmann et al., 2019). Such response modality-based task prioritization effects might be rooted in effector-based attentional weighting parameters that affect capacity scheduling schemes in simultaneous dual-task control (e.g., Logan & Gordon, 2001; see Huestegge & Koch, 2013, for details).

Based on this reasoning, one might speculate to what extent modality switch-cost asymmetries may become more likely with a decreasing temporal interval between trials, as this would principally reduce the opportunity for decay of effector-specific task-set representations. However, several previous observations speak against this idea. First, the study by Lawo and Koch (2015), which also involved auditory stimuli similar to our present study, explicitly manipulated the cue-stimulus interval (CSI, along with the response-stimulus interval, RSI) in a task requiring switches between vocal and manual responses (Experiment 3). However, they did not find any indication of switch-cost asymmetries regardless of CSI. Moreover, in the study that did report a significant switch-cost asymmetry for switching between vocal and manual responses (Philipp & Koch, 2011), the response-stimulus interval (RSI) was generally larger (around 1,600 ms) than in our current study (1,300 ms). In addition, the CSI was also varied but did not significantly modulate the switch-cost asymmetry. In fact, the switch-cost asymmetry in this study was numerically larger at long versus short CSIs (70 vs. 8 ms, respectively). Taken together, these observations render it unlikely that a reduction of the time intervals between trials may give rise to modality switch-cost asymmetries, probably because the decay of effector-related representations involved is too fast to matter in any situation not involving temporal task overlap.

### The role of modalities and modality mappings

One might argue that task-switching situations involving oculomotor responses are special. In particular, the lack of task dominance effects in task switching when one task involves oculomotor responses resembles a finding by Stephan et al. (2013), who did not observe any input-output modality compatibility effects when combining oculomotor and manual tasks triggered by visual and auditory stimuli. However, this was likely due to the fact that input-output modality compatibility effects in general are usually triggered mainly by the particular advantageous combination of auditory input with vocal output (see Hoffmann et al., 2019). In addition, the data by Stephan et al. (2013) mainly show that oculomotor responses can be triggered with comparable ease by both auditory and visual stimuli in task-switching settings. Thus, it appears unlikely that any special role of oculomotor responses can fully explain the present data pattern. Of course, one special characteristic of oculomotor responses is that they are usually executed faster and are more error-prone than other (manual, vocal) response types. However, these specific oculomotor characteristics did not prevent the occurrence of strong prioritization effects in simultaneous dual tasking (e.g., Hoffmann et al., 2019), and thus cannot explain the lack of similar effects in the present sequential task-processing paradigm.

Another difference between our present study and other, previous studies involving modality switching is related to the particular stimulus and cue modalities involved. For example, one might argue that a study by Lawo and Koch (2015) also involved auditory stimuli without finding response modality switch-cost asymmetries, whereas Philipp and Koch (2011) reported the emergence of such asymmetries in an experiment using visual stimuli. This might indicate that the presence of visual stimuli might be a crucial factor. However, another study by Philipp et al. (2013) that also involved visual stimuli failed to find significant response modality switch-cost asymmetries. Thus, the involvement of visual stimuli per se apparently does not suffice to yield response modality switch-cost asymmetries. In addition, previous research suggests that input-output modality compatibility effects only play a substantial role when different input-output modality mappings are intermixed within blocks of trials, not when stimulus (or response) modality is kept constant throughout a block of trials (e.g., Fintor et al., 2018; Lukas et al., 2010a), which was the case in our present study. Another, related issue might be cue modality: We here implemented visual cues (as in Lawo & Koch, 2015), and one might argue whether the involvement of auditory cues might have increased the chances of observing cost asymmetries. However, previous research indicates that the particular mapping of cue and stimulus modality has no substantial effect on main result patterns in task switching (e.g., Lukas et al., 2010b), but instead only yields general cue-modality switching effects (Koch et al., 2018). Taken together, it therefore appears unlikely that our present lack of response modality switch-cost asymmetries can simply be explained by our particular choice of cue or stimulus modalities.

### Mixing costs

Regarding mixing costs, we observed one single potentially interesting finding, namely *smaller* mixing costs for the oculomotor (vs. manual) task in Experiment 2. While we did not explicitly set up a priori hypotheses regarding mixing costs, it still appears noteworthy that this effect actually runs counter to what one would expect when transferring the logic of task dominance to asymmetric effects on task switching (see above). Probably, this particular effect in mixing costs can be explained by the overall lower RT level for saccades (vs. the other effector systems) in general. However, the numerical order of the effect sizes (largest mixing costs for manual, smallest costs for oculomotor) are generally in line with the response modality-based cost asymmetries observed in simultaneous dual tasking (Hoffmann et al., 2019). This could principally be interpreted in terms of potentially similar mechanisms (e.g., related to working memory updating) underlying mixing costs in task switching and dual-task costs in a simultaneous processing paradigm (Hirsch et al., 2018). In addition, our finding of large mixing costs in the auditory-manual pairing would also be in line with previous observations showing that modality-incompatible tasks are associated with larger mixing costs than modality-compatible tasks (Schacherer & Hazeltine, 2019), as these largest mixing costs were present in a modality-incompatible S-R pairing (auditory-manual). Nevertheless, despite these potentially plausible explanations, it should be kept in mind that the observed differences in mixing costs were quite small overall, and only seldom reached statistical significance.

## Conclusion

In sum, our data show that switching between two tasks that only differ in the associated response modality yielded typical mixing costs and switch costs similar to those observed in other task-switching situations, suggesting that response modality is an integral task set component. As we implemented three (instead of only two) different effector systems, different stimulus conditions, and much larger sample sizes compared to previous studies, these results substantially extend previous research. However, we found no clear indication of effector-based switch-cost asymmetries (between vocal and manual tasks, oculomotor and manual tasks, and oculomotor and vocal tasks) in task switching. This suggests that dual-task cost asymmetries based on effector systems (interpreted as oculomotor prioritization or vocal over manual prioritization) observed in simultaneous dual-task situations do not readily transfer to sequential task switching, probably due to different underlying representational dynamics of task set features. Consequently, even though more research is needed to further substantiate the lack of response modality-based switch-cost asymmetries, temporal task overlap might represent a prerequisite for the establishment of typical a priori capacity scheduling weights related to effector systems in multitasking.

## Data Availability

The data for all experiments are available at: 10.5281/zenodo.59205 31 . None of the experiments was preregistered. However, the general outline of the experiments was part of a previously approved DFG grant.

## Footnotes

1 Note that we observed a similar pattern when taking into account in which specific mixing block type (i.e., switching between oculomotor and vocal responses, between oculomotor and manual responses, or between vocal and manual responses) switch costs arose. Using this approach, manual and oculomotor switch costs were still both on a relatively similar low level, with manual switch costs amounting to 48 ms (when combined with an oculomotor task) and 58 ms (when combined with a vocal task), and oculomotor switch costs amounting to 56 ms (when combined with a manual task) and 60 ms (when combined with a vocal task), while vocal switch costs were descriptively greater, amounting to 73 ms and 78 ms (combined with an oculomotor or manual task, respectively).
